# Investigation of brain iron levels in Chinese patients with Alzheimer’s disease

**DOI:** 10.3389/fnagi.2023.1168845

**Published:** 2023-05-22

**Authors:** Chuanbin Huang, Jing Li, Chang Liu, Yong Zhang, Qiqiang Tang, Xinyi Lv, Mengyue Ruan, Kexue Deng

**Affiliations:** ^1^The First Affiliated Hospital of University of Science and Technology of China Anhui Provincial Hospital, Hefei, China; ^2^Fuyang Hospital of TCM, Fuyang, Anhui, China; ^3^Fuyang Hospital of Anhui Medical University, Fuyang, Anhui, China; ^4^GE HealthCare, Shanghai, China

**Keywords:** Alzheimer’s disease, mild cognitive impairment, quantitative susceptibility mapping, APOE, MRI

## Abstract

**Introduction:**

We aimed (i) to explore the diagnostic value of deep gray matter magnetic susceptibility in Alzheimer’s disease (AD) in China and (ii) to analyze its correlation with neuropsychiatric scales. Moreover, we conducted subgroup analysis based on the presence of the *APOE-*ε*4* gene to improve the diagnosis of AD.

**Methods:**

From the prospective studies of the China Aging and Neurodegenerative Initiative (CANDI), a total of 93 subjects who could undergo complete quantitative magnetic susceptibility imaging and *APOE-*ε*4* gene detection were selected. Differences in quantitative susceptibility mapping (QSM) values between and within groups, including AD patients, individuals with mild cognitive impairment (MCI), and healthy controls (HCs), both *APOE-*ε*4* carriers and non-carriers, were analyzed.

**Results:**

In primary analysis, the magnetic susceptibility values of the bilateral caudate nucleus and right putamen in the AD group and of the right caudate nucleus in the MCI group were significantly higher than those in the HCs group (*P* < 0.05). In *APOE-*ε*4* non-carriers, there were significant differences in more regions between the AD, MCI, and HCs groups, such as the left putamen and the right globus pallidus (*P* < 0.05). In subgroup analysis, the correlation between QSM values in some brain regions and neuropsychiatric scales was even stronger.

**Discussion:**

Exploration of the correlation between deep gray matter iron levels and AD may provide insight into the pathogenesis of AD and facilitate early diagnosis in elderly Chinese. Further subgroup analysis based on the presence of the *APOE-*ε*4* gene may further improve the diagnostic efficiency and sensitivity.

## Highlights

– The magnetic susceptibility of caudate nucleus and right putamen can diagnose Alzheimer’s disease.
– Further subgroup analysis revealed more differential brain regions.
– Significantly increased correlation with clinical scales after further subgroup analysis.

## 1. Introduction

Alzheimer’s disease (AD) is a genetically related neurodegenerative disease that is associated with impaired language, memory, and cognitive function. For many years, the *APOE-*ε*4* gene, which encodes an apolipoprotein, has been considered the most important genetic risk factor for AD pathogenesis. Its presence is highly correlated with AD (Huang et al., 2019; Serrano-Pozo et al., 2021). The association of *APOE* genes with senile plaques and neurofibrillary tangles has often been reported, but the relationship with brain iron content has not been reported.

Histochemical studies have shown that iron is the most abundant paramagnetic substance in the brain (Hare et al., 2013) and is involved in various brain activities such as neurotransmitter synthesis, oligodendrocyte differentiation, and myelination, as well as changes in mitochondrial function (Hallgren and Sourander, 1958; Cheli et al., 2021). Iron may play an important role in the pathogenesis of AD (Ayton et al., 2020). Excessive iron accumulation can lead to oxidative damage and cell death. Abnormal iron deposits are found in microglia, astrocytes, and senile plaques and around senile plaques and neurofibrillary tangles in AD patients (Connor et al., 1992; Smith et al., 1997; Rao and Adlard, 2018). Abnormally deposited iron will further promote beta-amyloid (Aβ) plaque formation and neurofibrillary tangles and accelerate disease progression (Liu et al., 2011; Teller et al., 2015; Ayton et al., 2017, 2018; Peters et al., 2018). This synergistic phenomenon seems to indicate that abnormal iron levels may have important implications for disease status assessment (van Bergen et al., 2016, 2018; O’Callaghan et al., 2017; Telling et al., 2017; Spotorno et al., 2020). However, Ayton et al. (2017) reported that in patients without Aβ deposition, iron deposition in the frontal lobe and caudate nucleus is associated with a decline in language function, whereas iron deposition in the hippocampus is associated with slightly improved cognitive function. Their study shows that dysregulation of iron content in different regions causes changes in cognitive function even without the effects of Aβ. Clinical experiments have shown that brain iron content intervention has a good alleviating effect on the decline of the self-care ability of AD patients (Crapper McLachlan et al., 1991). Therefore, efficient methods are needed to monitor the relationship between iron levels and AD.

Recently, it has been suggested that iron accumulation may be accurately measured *in vivo* using quantitative susceptibility mapping (QSM) (Zhu et al., 2009; Li et al., 2014; Liu et al., 2015; Kim et al., 2017; Du et al., 2018), a non-invasive magnetic resonance imaging (MRI) technique capable of inferring local tissue susceptibility secondary to the existence of iron (Langkammer et al., 2012; Zheng et al., 2013; Wang and Liu, 2015; Wang et al., 2017, 2022; Gong et al., 2019). In a longitudinal study, Damulina et al. (2020) found that throughout the brain, only the basal ganglia showed a significant increase in iron levels. White matter is rich in diamagnetic myelin and anisotropic fiber bundles, which can largely counteract the paramagnetic effects of iron. With QSM, the iron content can be better evaluated in deep gray matter than in white matter regions (Langkammer et al., 2012). Autopsy can only reveal final total iron levels, whereas QSM can be used to examine changes in magnetic susceptibility caused by long-term changes in iron levels, which may have important implications for disease prediction and progression. Several studies have only reported differences in iron levels between AD patients and those with mild cognitive impairment (MCI) (Zhu et al., 2009; Moon et al., 2016; Du et al., 2018; Guan et al., 2022; Yang et al., 2022; Kuchcinski et al., 2023). Therefore, the purpose of the present study was to investigate (i) the differences in deep gray matter magnetic susceptibility between *APOE-*ε*4* carriers and non-carriers in Chinese AD patients, MCI individuals, and healthy controls (HCs) and (ii) the correlation between deep gray matter magnetic susceptibility and MMSE and MoCA scores.

## 2. Materials and methods

### 2.1. Participants

This study was approved by the Institutional Ethics Committee. This is a retrospective study, from May 2019 to October 2021, subjects who participated in the single center prospective study of the China Aging and Neurodegenerative Initiative (CANDI) (Gao et al., 2022) and could undergo complete quantitative magnetic susceptibility imaging were selected for enrolled. All participants were evaluated by a trained physician with extensive experience in the diagnosis of dementia. Laboratory tests were conducted and the medical history was retrieved. All participants filled in the Mini-Mental State Examination (MMSE), Montreal Cognitive Assessment scale (MoCA), and Clinical Dementia Rating Scale (CDR) questionnaires for quick assessment of their cognitive ability. Finally, MRI analysis and *APOE* genotype determination were performed. The main inclusion criteria were as follows. All subjects were 40–80 years old and had no contraindications for MRI. AD and MCI patients or their families had confirmed cognitive decline. Patients were diagnosed with AD according to the National Institute on Aging Alzheimer’s Association 2011 (NIA-AA 2011) AD dementia core clinical criteria (McKhann et al., 2011). The MCI diagnosis was made based on the Peterson criteria (Petersen et al., 2018). Exclusion criteria for this study include individuals with other types of dementia, a medical history that may cause neurological diseases, previous brain injury or psychiatric disorders, and poor image quality. Additionally, all subjects were enrolled based on a comprehensive evaluation using the A/T/N framework detection system. This framework comprises three components: “A” for Aβ deposition, which can be detected through positron emission tomography (PET) or cerebrospinal fluid (CSF) Aβ42/Aβ40 ratios; “T” for aggregated tau, which can be detected through PET tau or CSF phosphorylated tau; and “N” for neuronal degeneration or injury, which can be detected through 18-fluorodeoxyglucose-PET and structural magnetic resonance imaging (Gao et al., 2022).

A total of 93 subjects were enrolled, comprising 43 AD patients (29 *APOE-*ε*4* carriers and 14 non-carriers), 23 MCI patients (10 *APOE-*ε*4* carriers and 13 non-carriers), and 27 age-matched HCs (4 *APOE-*ε*4* carriers, 22 non-carriers, from which one subject was excluded due to lack of *APOE-*ε*4* gene data in further analysis) with no history of neurological injury.

### 2.2. MRI and *APOE* gene detection

The QSM scans were made with a 3.0-T MR750w scanner using a head and neck coil (GE Healthcare, Milwaukee, WI, USA). Earplugs and foam padding were used to reduce scanner noise and head movements. Imaging parameters were as follows: repetition time = 31.4 ms, flip angle = 12°, field of view = 25.6 cm, matrix size = 256 × 256, acceleration factor = 2, number of echoes = 12, first echo time = 2.0 ms, echo time spacing = 2.35 ms, slice number = 136, the total acquisition time = 7 min.

For *APOE* gene detection, all subjects fasted for>8 h, and 3 ml of venous blood was collected from the cubital vein and placed in dipotassium ethylenediaminetetraacetic anticoagulant tubes at room temperature. The *APOE* gene was detected within 2 h by PCR.

### 2.3. Image processing and analysis

Images were processed as previously described (Li et al., 2011). Images were reconstructed referring to the mean susceptibility value of the whole brain based on the Larmor frequency. Then the spherical average method was used to remove the background phase and set the filter radius to 8 (Schweser et al., 2011). The improved Laplacian phase unwrapping method and temporal fitting were used (Li et al., 2011, 2015) and the regularization threshold for Laplace filtering was set to 0.04 to obtain the susceptibility map from the brain tissue frequency map.

The QSM values for each deep gray matter nucleus were gained from all visible areas. Regions of interest (ROIs) included the bilateral caudate nucleus (CN), putamen (PUT), globus pallidus (GP), substantia nigra (SN), and red nucleus (RN). All ROIs were manually outlined on all consecutive levels using MRIcro software^1^ by one neuroradiologist who was blinded to the subject group. QSM values were measured again after 2 and 4 weeks, and the average value was taken. To confirm the reliability of the results, 25 subjects were randomly selected from all subjects with post-processed QSM images, and their ROIs were manually outlined on all consecutive levels by another neuroradiologist (>16 years) using the same method.

### 2.4. Statistical analysis

Data were statistically analyzed using SPSS (SPSS version 22.0, SPSS, Chicago, IL, USA) and GraphPad software (GraphPad Prism version 8.0, GraphPad Software Inc., San Diego, CA, USA). One-way ANOVA was conducted for normally distributed data with homogeneity of variance. The Kruskal–Wallis test was used for non-normally distributed data. We further conducted Bonferroni correction analysis to make comparisons between the AD and HCs, MCI and HCs, and AD and MCI groups. QSM values were compared between the *APOE-*ε*4* carrier and non-carrier subgroups in the AD, MCI, and HCs groups, and between males and females within groups using the independent samples *t*-test. ROC curve analysis was conducted to evaluate the sensitivity and specificity of the QSM values in the three groups. Between-group gender analysis was conducted using the chi-square test. *P* < 0.05 was considered statistically significant. Partial correlation analysis of magnetic susceptibility in three groups was performed based on the MMSE and MoCA scores in different brain regions after adjusting for age and gender. Considering the small number of HCs in the *APOE-*ε*4* carrier group, they were excluded from the within group gender analysis and partial correlation analysis.

## 3. Results

### 3.1. Clinical data

Interobserver variability was analyzed based on the intraclass correlation coefficient. The results show satisfactory consistency (0.815–0.941) (Table 1).

**TABLE 1:** Results of analysis of interobserver agreement.

ROIs	Left hemibrain ICC (95% CI)	Right hemibrain ICC (95% CI)
Caudate nucleus	0.850(0.671−0.933)	0.815(0.610−0.916)
Putamens	0.934(0.693−0.978)	0.921(0.766−0.969)
Globus pallidus	0.863(0.716−0.937)	0.904(0.790−0.957)
Substantia nigra	0.906(0.800−0.957)	0.894(0.777−0.952)
Red nucleus	0.933(0.834−0.971)	0.941(0.826−0.977)

Ninety-three patients (61.81 ± 8.46, 30 men) were evaluated. The gender ratio (men 13/7/10, women 30/16/17, *P* = 0.820), age (62.63 ± 8.10, 63.91 ± 7.77, 58.70 ± 8.99, *P* = 0.077), and educational level (8.98 ± 4.07, 8.91 ± 4.69, 11.04 ± 3.86, *P* = 0.099) were not significantly different between the three groups. As expected, the MMSE and MoCA scores were ordered as AD < MCI < HCs, and the CDR scores were ordered as AD > MCI > HCs (*P* < 0.05) (Table 2).

**TABLE 2:** Demographic and neuropsychological scores.

Variable (mean ± SD)	AD	MCI	HCs	χ^2^ value	*P*-value
***APOE-*ε *4* gene not considered**					
Gender (male/female)	43(13/30)	23(7/16)	27(10/17)	0.398	0.820
Age (year)	62.63 ± 8.10	63.91 ± 7.77	58.70 ± 8.99	5.121	0.077
Education (year)	8.98 ± 4.07	8.91 ± 4.69	11.04 ± 3.86	4.622	0.099
MMSE	15.72 ± 6.55	20.83 ± 5.47	27.30 ± 2.20	48.626	0.000**
MOCA	11.68 ± 6.15	16.00 ± 6.42	23.08 ± 3.91	41.286	0.000**
CDR	1.00 ± 0.42	0.50 ± 0.00	0.10 ± 0.24	66.926	0.000**
***APOE-*ε *4* non-carriers**					
Gender (male/female)	14(4/10)	13(4/9)	22(6/16)	0.049	0.976
Age (year)	63.64 ± 7.82	62.92 ± 8.42	58.00 ± 7.28	5.748	0.056
Education (year)	9.29 ± 4.14	8.77 ± 4.92	10.96 ± 4.04	2.168	0.338
MMSE	16.21 ± 6.42	21.69 ± 5.56	27.32 ± 2.30	27.690	0.000**
MOCA	11.76 ± 5.41	17.54 ± 6.62	23.23 ± 3.82	24.686	0.000**
CDR	1.00 ± 0.48	0.50 ± 0.00	0.10 ± 0.25	34.917	0.000**
***APOE-*ε *4* carriers**					
Gender (male/female)	29(9/20)	10(3/7)	4(3/1)	3.128	0.209
Age (year)	62.14 ± 8.32	65.20 ± 7.07	59.25 ± 16.32	0.800	0.670
Education (year)	8.83 ± 4.10	9.10 ± 4.63	12.00 ± 3.46	2.580	0.275
MMSE	15.48 ± 6.72	19.70 ± 5.44	28.00 ± 0.82	11.720	0.003**
MOCA	11.64 ± 6.57	14.00 ± 5.87	22.00 ± 5.35	6.954	0.031*
CDR	1.00 ± 0.40	0.50 ± 0.00	0.13 ± 0.25	24.401	0.000**

*Indicates significant difference *p* < 0.05. **Indicates significant difference *p* < 0.01. AD, Alzheimer’s disease; MCI, mild cognitive impairment; HCs, healthy controls; MMSE, mini-mental state examination; MoCA, montreal cognitive assessment scale; CDR, clinical dementia rating scale.

### 3.2. Comparison of QSM values among three groups

**FIGURE 1:**
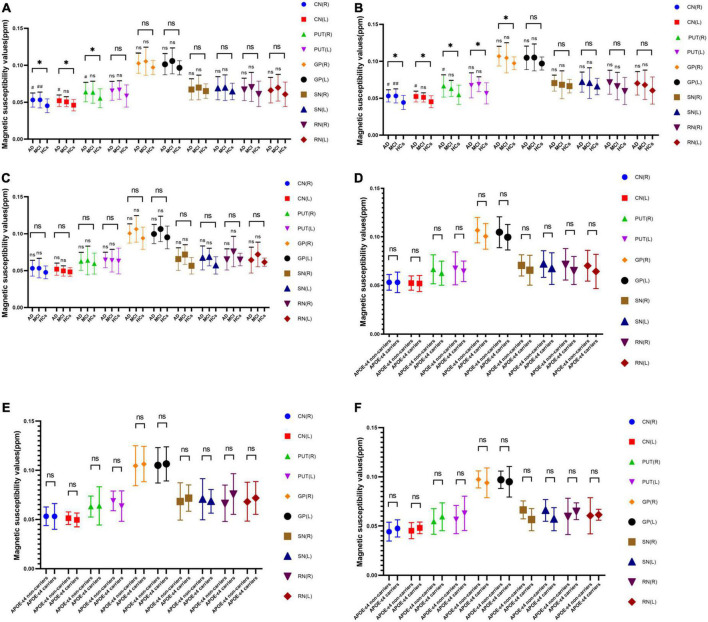
Without *APOE*-ε4 consideration **(A)**, APOE-ε4 non-carriers **(B)**, and APOE-ε4 carriers **(C)**. Statistical analysis of QSM values of each group. *APOE-*ε*4* gene of AD **(D)**, MCI **(E)**, and HCs **(F)** groups. Statistical analysis of QSM values between non-carriers and carriers. *Indicates that there is significant difference among the AD, MCI, and HCs groups by one-way ANOVA (*p* < 0.05). ^#^ and ^##^ indicate that there is significant difference between the AD and HCs groups, MCI and HCs groups by Bonferroni Correction Analysis (*P* < 0.05). AD, Alzheimer’s disease. MCI; mild cognitive impairment; HCs, Healthy controls; ppm, parts per million.

In all subjects, there were statistically significant differences in QSM values of the bilateral caudate nucleus and right putamen between the AD, MCI, and HCs groups (Figure 1A and Table 3). Among *APOE-*ε*4* non-carriers, QSM values in the bilateral caudate nucleus, bilateral putamen, and right globus pallidus were significantly different between the AD, MCI, and HCs groups (Figure 1B and Table 3). No significant difference in QSM values was observed among *APOE-*ε*4* carriers (Figure 1C and Table 3).

**TABLE 3:** Comparison of QSM values for different ROIs between groups (ppm).

Variable (mean ± SD)	AD	MCI	HCs	*P-*value
***APOE-*ε *4* gene not considered**				
Caudate nucleus (R)	0.053 ± 0.0097^#^	0.053 ± 0.0109^##^	0.045 ± 0.0093	0.003**
Caudate nucleus (L)	0.052 ± 0.0079^#^	0.050 ± 0.0066	0.046 ± 0.0077	0.006**
Putamen (R)	0.064 ± 0.0132^#^	0.063 ± 0.0147	0.055 ± 0.0128	0.030*
Putamen (L)	0.065 ± 0.0129	0.067 ± 0.0127	0.058 ± 0.0150	0.059
Globus pallidus (R)	0.103 ± 0.0134	0.105 ± 0.0190	0.097 ± 0.0095	0.060
Globus pallidus (L)	0.101 ± 0.0142	0.106 ± 0.0173	0.097 ± 0.0096	0.113
Substantia nigra (R)	0.067 ± 0.0141	0.070 ± 0.0165	0.065 ± 0.0098	0.473
Substantia nigra (L)	0.069 ± 0.0155	0.070 ± 0.0173	0.064 ± 0.0112	0.402
Red nucleus (R)	0.067 ± 0.0152	0.071 ± 0.0196	0.061 ± 0.0172	0.143
Red nucleus (L)	0.066 ± 0.0171	0.070 ± 0.0182	0.061 ± 0.0166	0.176
***APOE-*ε *4* non-carriers**				
Caudate nucleus (R)	0.053 ± 0.0081^#^	0.053 ± 0.0095^##^	0.044 ± 0.0095	0.006**
Caudate nucleus (L)	0.052 ± 0.0074^#^	0.051 ± 0.0064	0.045 ± 0.0081	0.015*
Putamen (R)	0.066 ± 0.0149^#^	0.063 ± 0.0107	0.055 ± 0.0130	0.026*
Putamen (L)	0.067 ± 0.0170	0.069 ± 0.0100	0.057 ± 0.0143	0.024*
Globus pallidus (R)	0.107 ± 0.0132	0.105 ± 0.0204	0.097 ± 0.0087	0.031*
Globus pallidus (L)	0.105 ± 0.0160	0.105 ± 0.0180	0.097 ± 0.0089	0.224
Substantia nigra (R)	0.071 ± 0.0109	0.068 ± 0.0190	0.066 ± 0.0092	0.288
Substantia nigra (L)	0.072 ± 0.0137	0.071 ± 0.0209	0.066 ± 0.0111	0.334
Red nucleus (R)	0.072 ± 0.0162	0.066 ± 0.0185	0.060 ± 0.0184	0.155
Red nucleus (L)	0.070 ± 0.0158	0.068 ± 0.0198	0.060 ± 0.0183	0.245
***APOE-*ε *4* carriers**				
Caudate nucleus (R)	0.053 ± 0.0110	0.053 ± 0.0130	0.048 ± 0.0090	0.637
Caudate nucleus (L)	0.052 ± 0.0080	0.050 ± 0.0070	0.048 ± 0.0060	0.537
Putamen (R)	0.063 ± 0.0120	0.064 ± 0.0190	0.059 ± 0.0140	0.879
Putamen (L)	0.064 ± 0.0110	0.064 ± 0.0160	0.063 ± 0.0170	0.964
Globus pallidus (R)	0.100 ± 0.0130	0.106 ± 0.0180	0.094 ± 0.0150	0.324
Globus pallidus (L)	0.100 ± 0.0130	0.107 ± 0.0170	0.095 ± 0.0160	0.308
Substantia nigra (R)	0.066 ± 0.0150	0.072 ± 0.0130	0.057 ± 0.0110	0.214
Substantia nigra (L)	0.067 ± 0.0160	0.068 ± 0.0120	0.057 ± 0.0120	0.414
Red nucleus (R)	0.065 ± 0.0140	0.076 ± 0.0210	0.065 ± 0.0090	0.189
Red nucleus (L)	0.064 ± 0.0180	0.072 ± 0.0170	0.061 ± 0.0060	0.441

Values are in mean ± standard deviation. *Indicates significant difference *p* < 0.05. **Indicates significant difference *p* < 0.01. ^#^ and ^##^ Indicate that there is significant difference between the AD and HCs groups, MCI and HCs groups by Bonferroni correction analysis (*p* < 0.05). AD, Alzheimer’s disease; MCI, mild cognitive impairment; HCs, healthy controls; ppm, parts per million; R, Right; L, Left.

### 3.3. Comparison of QSM values between two groups

The QSM values of the bilateral caudate and right putamen in the AD group and of the right caudate nucleus in the MCI group were significantly higher than those in the HCs group (Figure 1B and Table 3). Among *APOE-*ε*4* non-carriers, QSM values of the bilateral caudate and right putamen in the AD group and of the right caudate nucleus in the MCI group were significantly higher than those in the HCs group (Figure 1B and Table 3). No significant differences were observed in QSM values between the AD and MCI groups for *APOE-*ε*4* non-carriers or between the AD, MCI, and HCs groups for *APOE-*ε*4* carriers (Figures 1B, C and Table 3).

Within the AD, MCI, and HCs groups, no significant differences in QSM values were observed between *APOE-*ε*4* carriers and non-carriers (Figures 1D–F).

### 3.4. Comparison of QSM values between genders within groups

**FIGURE 2:**
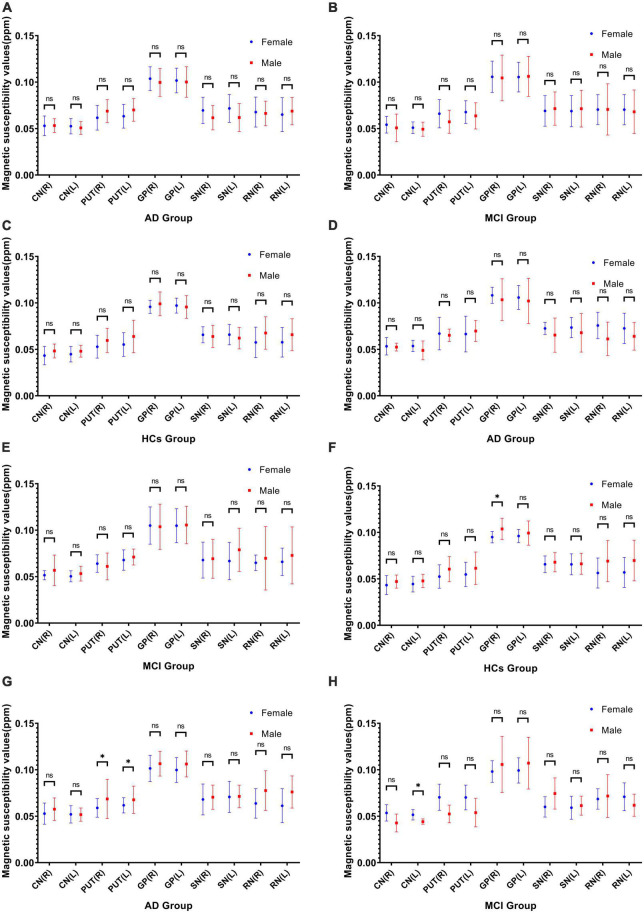
Not considering *APOE-*ε*4* gene comparison of QSM values between genders within three groups: AD **(A)**, MCI **(B)**, and HCs **(C)**. *APOE-*ε*4* non-carriers comparison of QSM values between genders within three groups: AD **(D)**, MCI **(E)**, and HCs **(F)**. *APOE-*ε*4* carriers comparison of QSM values between genders within AD **(G)** and MCI **(H)** groups. * Indicates that there is significant difference by independent samples *t*-test (*P* < 0.05). CN, caudate nucleus; PUT, putamen; GP, globus pallidus; SN; substantia nigra; RN, red nucleus; R, right; L, left; ppm, parts per million.

Among *APOE-*ε*4* non-carriers, there was a significant gender differences in QSM values of the right pallidus (95% CI: -0.017 to -0.001; *P* = 0.030) in the HCs group (Figure 2F). Among *APOE-*ε*4* carriers, there were significant gender differences in QSM values of the bilateral putamen (Right; 95% CI: -0.0208 to -0.0021; *P* = 0.019), (Left; 95% CI: -0.0166 to -0.0002; *P* = 0.045) in the AD group (Figure 2G) and of the left caudate nucleus (95% CI: 0.0003 to 0.0148; *P* = 0.044) in the MCI group (Figure 2H). No significant differences were observed in QSM values of different genders between the AD and MCI groups for *APOE-*ε*4* non-carriers or between the AD, MCI, and HCs groups for *APOE-*ε*4* gene not considered (Figures 2A–E).

### 3.5. ROC curve analysis

**FIGURE 3:**
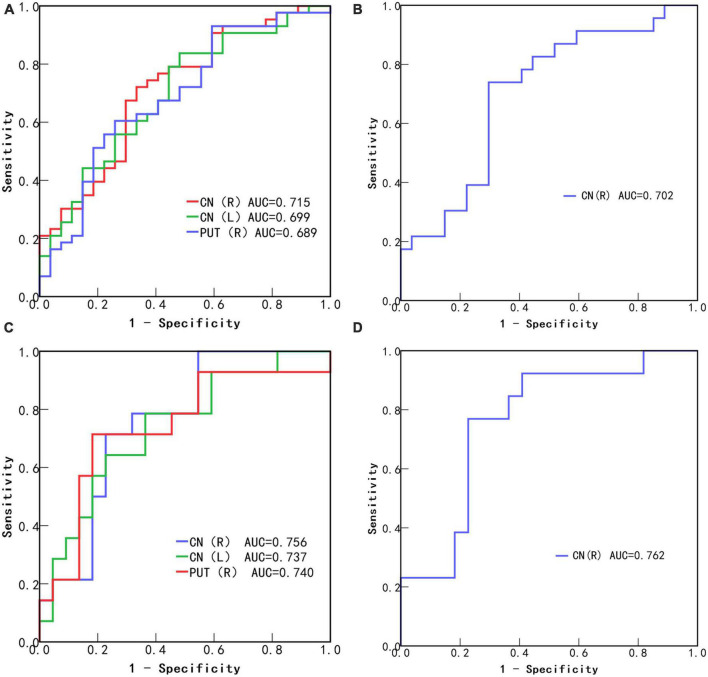
Receiver operating characteristic (ROC) curves of statistically significant brain region QSM values to diagnose Alzheimer’s disease **(A)** and mild cognitive impairment **(B)** without considering *APOE-*ε*4*. ROC curves of statistically significant brain region QSM values to diagnose Alzheimer’s disease **(C)** and mild cognitive impairment **(D)** in APOE-ε4 non-carriers. CN, caudate nucleus; PUT, putamen; R, Right; L, Left.

In all subjects, the area under the ROC curve (AUC) values of the bilateral caudate nucleus and the right putamen in the AD group were (Right caudate nucleus; AUC, 0.715; 95% CI: 0.59–0.84; *P* = 0.003), (Left caudate nucleus; AUC, 0.699; 95% CI: 0.57–0.82; *P* = 0.005), and (Right putamen; AUC, 0.689; 95% CI: 0.56–0.82; *P* = 0.008) (Figure 3A), and the AUC value of the right caudate nucleus in the MCI group was (AUC, 0.702; 95% CI: 0.56–0.85; *P* = 0.015) (Figure 3B). In *APOE-*ε*4* non-carriers, the *AUC* values of the bilateral caudate nucleus and the right putamen in the AD group were (Right caudate nucleus; AUC, 0.756; 95% CI: 0.60–0.91; *P* = 0.010), (Left caudate nucleus; AUC, 0.737; 95% CI: 0.57–0.91; *P* = 0.018), and (Right putamen; AUC, 0.74; 95% CI: 0.56–0.92; *P* = 0.016) (Figure 3C), and the AUC value of the right caudate nucleus in the MCI group was (AUC, 0.762; 95% CI: 0.60–0.93; *P* = 0.010) (Figure 3D).

### 3.6. Correlation analysis

**FIGURE 4:**
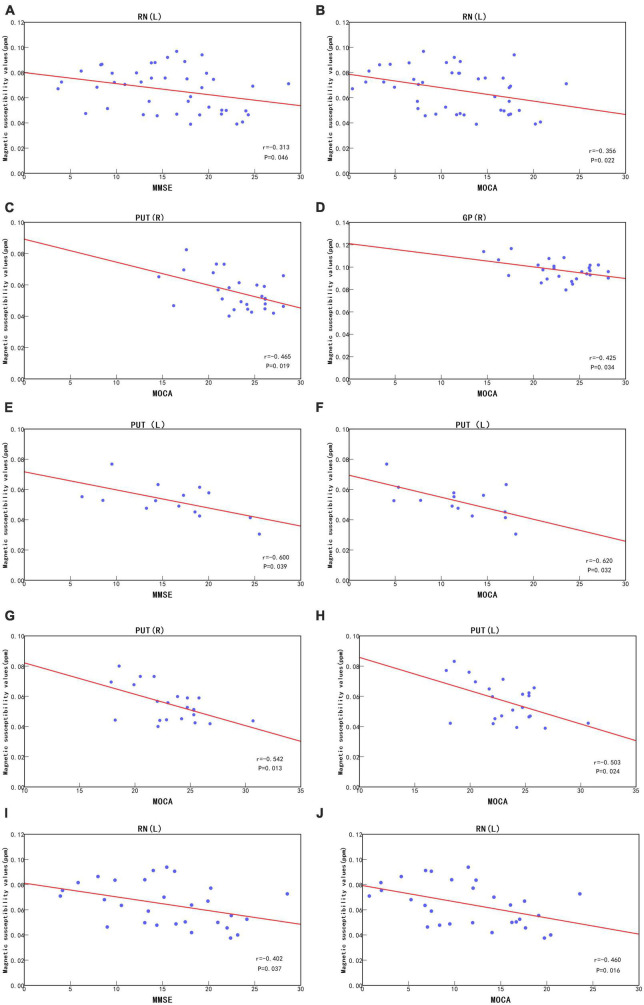
Under no consideration of *APOE-*ε*4*, correlation analysis between left red nucleus QSM values and MMSE scores **(A)** and MOCA scores **(B)** in the AD group and correlation analysis between right putamen and right globus pallidus QSM values and MOCA scores **(C,D)** in the HCs group. In *APOE-*ε*4* non-carriers, correlation analysis between left putamen QSM values and MMSE scores **(E)** and MOCA scores **(F)** in the AD group and correlation analysis between bilateral putamen QSM values and MOCA scores **(G,H)** in the HCs group. In *APOE*-ε4 carriers, correlation analysis between left red nucleus QSM values and MMSE scores **(I)** and MOCA scores **(J)** in the AD group. PUT, putamen; RN, red nucleus; GP, globus pallidus; R, Right; L, Left; ppm, parts per million.

For all subjects, left red nucleus QSM values in the AD group was negatively correlated with MMSE (*r* = -0.313, *P* = 0.046) and MoCA scores (*r* = -0.356, *P* = 0.022), and right putamen (*r* = -0.465, *P* = 0.019) and right globus pallidus (*r* = -0.425, *P* = 0.034) QSM values in HCs groups was negatively correlated with MoCA scores (Figures 4A–D).

In *APOE-*ε*4* non-carriers, QSM values of the left putamen in the AD group were negatively correlated with the MMSE (*r* = -0.600, *P* = 0.039) and MoCA scores (*r* = -0.620, *P* = 0.032), and QSM values in the bilateral putamen in the HCs group were negatively correlated with MoCA scores (Right, *r* = -0.542, *P* = 0.013) (Left, *r* = -0.503, *P* = 0.024) (Figures 4E–H). In *APOE-*ε*4* carriers, QSM values of the left red nucleus in the AD group were negatively correlated with the MMSE (*r* = -0.402, *P* = 0.037) and MoCA scores (*r* = -0.460, *P* = 0.016) (Figures 4I, J). In *APOE-*ε*4* carriers and non-carriers in the MCI group, QSM values were not significantly correlated with the MMSE and MoCA scores (*P* > 0.05).

## 4. Discussion

In the present study, we examined the differences in magnetic susceptibility in deep gray matter in the AD, MCI, and HCs groups, including both *APOE-*ε*4* carriers and non-carriers, and their correlation with cognitive performance. Changes in deep gray matter magnetic susceptibility in *APOE-*ε*4* carriers and non-carriers were further investigated in the AD and MCI groups, and its correlation with neuropsychiatric scales was analyzed.

As with previous reports (Bailly et al., 2019; Rajan et al., 2021; Alzheimer’s Association, 2023), the number of female patients in our study was notably higher than that of males. Our findings align with the van der Weerd study (van der Weerd et al., 2022), showing no significant gender differences within the AD, MCI, and HCs groups. It is important to highlight that in further analysis of *APOE-*ε*4* gene subgroups, there were significant differences in QSM values among various brain regions and gender groups. Specifically, in HCs without *APOE-*ε*4*, the right pallidus had higher QSM values in males compared to females, while in individuals with AD and *APOE-*ε*4*, the bilateral putamen had higher QSM values in males compared to females. Additionally, in individuals with MCI and the *APOE-*ε*4*, the left caudate nucleus had higher QSM values in females compared to males. Currently, no other comparable results have been discovered. It remains uncertain whether this outcome is indicative of a more nuanced subgroup analysis or if it is potentially biased due to a smaller sample size. Therefore, additional research is necessary to identify the underlying cause of this result.

We found that the *APOE-*ε*4* gene may have potential effects on iron levels. When the *APOE-*ε*4* gene was not considered, there were significant differences in magnetic susceptibility between the bilateral caudate nucleus and right putamen among the AD, MCI, and HCs groups, consistent with Moon’s discovery of abnormal iron deposition in the caudate and putamen (Moon et al., 2016; Guan et al., 2022). Compared with the HCs group, the QSM values of the bilateral caudate nucleus and the right putamen in the AD group and of the right caudate nucleus in the MCI group were significantly increased. It is worth noting that more brain regions showed an increase in magnetic susceptibility in the AD group than in the MCI group, suggesting that iron levels may gradually increase with disease progression. This is partially consistent with other reported results (Zhu et al., 2009; Du et al., 2018; Guan et al., 2022). In *APOE-*ε*4* non-carriers, we not only found similar results, but we also observed significant differences in QSM values for the left putamen and right pallidus. Whether there are more regions with changes in iron content requires further study. However, the same results were not observed in *APOE-*ε*4* carriers, possibly because of bias caused by the small number of subjects in the HCs group. An increase in brain iron content in multiple regions in AD and MCI patients compared to the HCs group could be observed, although these differences were not statistically significant. Although the QSM values in some brain regions of *APOE-*ε*4* carriers were slightly increased compared with those of *APOE-*ε*4* non-carriers, no statistically significant differences were observed. This contradicts the results reported by Yim that *APOE-*ε*4* carry leads to elevated iron levels (Yim et al., 2022) but is consistent with most of the findings reported by van Berge (van Bergen et al., 2016). A reason for this discrepancy may be that in other studies, only one or a few slices of the ROI were selected, while we selected all consecutive slices of the ROI.

Our data suggest that changes in magnetic susceptibility can be used to distinguish between different groups. The preliminary analysis results showed that, among all subjects, QSM values in the bilateral caudate nucleus and right putamen showed the highest changes in the AD group. More importantly, in the early MCI stage of the disease, the QSM value of the right caudate nucleus showed the strongest changes. Further subgroup analysis based on the presence of the *APOE-*ε*4* gene further improved the diagnostic specificity and diagnostic performance. However, the AUC values obtained did not surpass 0.8, indicating that the diagnostic efficacy of this biomarker may not be optimal. Further studies may require a larger sample size, selection of additional regions of interest, or utilization of multimodal imaging techniques to enhance diagnostic performance. In *APOE-*ε*4* non-carriers, local iron levels were more strongly associated with cognitive changes, providing more clues for the prediction and diagnosis of AD and MCI. We observed significant differences in more brain regions, such as the left putamen and right globus pallidus, between the AD, MCI, and HCs groups in *APOE-*ε*4* non-carriers. However, no differences were observed when the AD and MCI groups were compared with the HCs group, possibly because of the small sample size. Further research is required to expand the sample size.

In all subjects, we observed weak correlations between (i) the QSM values of the left red nucleus in the AD group and the right putamen and right globus pallidus in the HCs group and (ii) the neuropsychiatric scale scores. We observed no significant correlations for other brain regions. This is consistent with the findings of Moon et al. (2016) and Yim et al. (2022). However, Du et al. (2018) reported a correlation between the left caudate nucleus and scale scores in the AD group. AD and MCI patients in the present study had significantly lower MMSE and MoCA scores than in the study by Du et al. (2018), possibly because Du selected mild-to-moderate patients with relatively good cognitive function, whereas cognitive function was relatively poor in the present study. Although QSM values were significantly elevated in the AD and MCI groups, there was no significant correlation between magnetic susceptibility of the caudate nucleus and cognitive scale scores. The basal ganglia play an important role in learning and cognitive processes (Choi et al., 2020), but significant correlations between clinical scales and iron levels in other brain regions have not been observed. This lack of significant correlation could be due to several reasons. First, despite changes in cognitive function (Ayton et al., 2017, 2018), we only used MMSE and MoCA as cognitive impairment assessment criteria, which may not reflect the cognitive status of the patients in detail. Second, factors such as neuroinflammation can also lead to increases in iron levels (Ayton et al., 2020), and a significant increase in iron levels only reflects neurodegeneration, not cognitive decline. Third, even a significant increase in iron levels does not result in cognitive decline. Interestingly, subgroup analysis based on the presence of the *APOE-*ε*4* gene revealed a weak correlation between the QSM value of the left red nucleus and neuropsychiatric scale scores in the AD group only in *APOE-*ε*4* carriers. Among *APOE-*ε*4* non-carriers, there was a strong correlation between (i) QSM values in the left putamen in the AD group and the bilateral putamen in the HCs group and (ii) neuropsychiatric scale scores. Our research has certain limitations. Due to the small sample size of this study, the ROI only included deep gray matter nuclei, so other disease-related ROIs might have been excluded. Moreover, the presence of substances such as copper, zinc, and myelin in the brain may has a certain effect on magnetic susceptibility. We conducted only a horizontal comparative study with a limited sample size, so more data are needed to investigate the relationship between AD progression and magnetic susceptibility.

Although iron levels in AD and MCI patients have been reported, we investigated iron levels in the AD, MCI, and HCs groups based on the presence of the *APOE-*ε*4* gene. Expanding on previous studies, we revealed that the crucial role of identifying MCI specifically in the right caudate nucleus, and the detecting AD in both the bilateral caudate nucleus and the right putamen nucleus is equally critical.

## Data availability statement

The data analyzed in this study is subject to the following licenses/restrictions: the raw data supporting the conclusions of this article will be made available by the authors, without undue reservation. Requests to access these datasets should be directed to KD, dengkexue-anhui@163.com.

## Ethics statement

The studies involving human participants were reviewed and approved by the Medical Research Ethics Committee of the First Affiliated Hospital of the University of Science and Technology of China. Written informed consent for participation was not required for this study in accordance with the national legislation and the institutional requirements. Written informed consent was not obtained from the individual (s) for the publication of any potentially identifiable images or data included in this article.

## Author contributions

CH, JL, and KD contributed to conception and design of the study. CL, QT, XL, and MR organized the database. CH and YZ performed the statistical analysis. CH and JL wrote the first draft of the manuscript. CL, YZ, QT, XL, and MR wrote sections of the manuscript. All authors contributed to manuscript revision, read, and approved the submitted version.

## Footnotes

1
http://www.mricro.com
